# Genomic evaluation for a three-way crossbreeding system considering breed-of-origin of alleles

**DOI:** 10.1186/s12711-017-0350-1

**Published:** 2017-10-23

**Authors:** Claudia A. Sevillano, Jeremie Vandenplas, John W. M. Bastiaansen, Rob Bergsma, Mario P. L. Calus

**Affiliations:** 1 Wageningen University & Research Animal Breeding and Genomics, 6700 AH Wageningen, The Netherlands; 2 Topigs Norsvin Research Center, 6640 AA Beuningen, The Netherlands

## Abstract

**Background:**

Genomic prediction of purebred animals for crossbred performance can be based on a model that estimates effects of single nucleotide polymorphisms (SNPs) in purebreds on crossbred performance. For crossbred performance, SNP effects might be breed-specific due to differences between breeds in allele frequencies and linkage disequilibrium patterns between SNPs and quantitative trait loci. Accurately tracing the breed-of-origin of alleles (BOA) in three-way crosses is possible with a recently developed procedure called BOA. A model that accounts for breed-specific SNP effects (BOA model), has never been tested empirically on a three-way crossbreeding scheme. Therefore, the objectives of this study were to evaluate the estimates of variance components and the predictive accuracy of the BOA model compared to models in which SNP effects for crossbred performance were assumed to be the same across breeds, using either breed-specific allele frequencies (${\text{G}}_{\text{A}}$ model) or allele frequencies averaged across breeds (${\text{G}}_{\text{B}}$ model). In this study, we used data from purebred and three-way crossbred pigs on average daily gain (ADG), back fat thickness (BF), and loin depth (LD).

**Results:**

Estimates of variance components for crossbred performance from the BOA model were mostly similar to estimates from models ${\text{G}}_{\text{A}}$ and ${\text{G}}_{\text{B}}$. Heritabilities for crossbred performance ranged from 0.24 to 0.46 between traits. Genetic correlations between purebred and crossbred performance (${\text{r}}_{\text{pc}}$) across breeds ranged from 0.30 to 0.62 for ADG and from 0.53 to 0.74 for BF and LD. For ADG, prediction accuracies of the BOA model were higher than those of the ${\text{G}}_{\text{A}}$ and ${\text{G}}_{\text{B}}$ models, with significantly higher accuracies only for one maternal breed. For BF and LD, prediction accuracies of models ${\text{G}}_{\text{A}}$ and ${\text{G}}_{\text{B}}$ were higher than those of the BOA model, with no significant differences. Across all traits, models ${\text{G}}_{\text{A}}$ and ${\text{G}}_{\text{B}}$ yielded similar predictions.

**Conclusions:**

The BOA model yielded a higher prediction accuracy for ADG in one maternal breed, which had the lowest ${\text{r}}_{\text{pc}}$ (0.30). Using the BOA model was especially relevant for traits with a low ${\text{r}}_{\text{pc}}$. In all other cases, the use of crossbred information in models ${\text{G}}_{\text{A}}$ and ${\text{G}}_{\text{B}}$, does not jeopardize predictions and these models are more easily implemented than the BOA model.

**Electronic supplementary material:**

The online version of this article (doi:10.1186/s12711-017-0350-1) contains supplementary material, which is available to authorized users.

## Background

Genomic selection (GS) is more accurate than pedigree-based selection, and thus was developed for purebred (PB) populations of many farm species [1–4]. However, many production systems use crossbreeding schemes to produce crossbred (CB) individuals for commercial production. Crossbreeding in plants is common practice in many crops, such as maize. Crossbreeding in animals is common practice for pigs and poultry, and, in cattle, the use of crosses or composite breeds contributes largely to the beef and dairy industry. If selection is based on the performance measured on PB individuals, the rate of genetic change observed in CB individuals may be reduced because of differences in additive variance between PB and CB individuals, and because the genetic correlation between performance in PB and CB individuals (${\text{r}}_{\text{pc}}$) is lower than 1 [5, 6]. With ${\text{r}}_{\text{pc}}$ values of 0.7 or lower, using only PB performance was predicted to yield considerably less genetic progress in CB performance compared to using performance of both PB and CB [7, 8]. In pigs, ${\text{r}}_{\text{pc}}$ lower than 0.7 were reported for daily gain, daily feed intake, feed conversion ratio and residual feed intake [9–11], and also in poultry for egg number [12], and in cattle for weight-related traits [13]. In maize, the correlation between PB and CB performance for grain yield (GY) is lower than that for grain dry matter content (GDMC), and it was observed that models that do not include CB information failed to predict the performance of CB for GY but for GDMC yielded a high prediction accuracy [14].

With GS, training with CB information is facilitated because GS eliminates the disadvantages of having to record pedigree data on CB individuals [7]. Moreover, GS using CB information could benefit from models that estimate the effects on CB performance of markers that segregate within the parental breeds, as suggested by Dekkers [7], Ibáñez-Escriche et al. [15], Kinghorn et al. [16] and Christensen et al. [17, 18] in the context of animal breeding, and by Schrag et al. [14] in the context of hybrid performance in maize.

A commonly used GS model, known as genomic best linear unbiased prediction (GBLUP) [19], replaces the pedigree-based relationship matrix by a genomic relationship matrix. The values in the genomic relationship matrix are a function of allele content and allele frequencies [20]. Consequently, the genomic relationship matrix is built under the assumption that all individuals belong to the same population, with the same average allele contents. Moreover, GBLUP implicitly assumes a single value for the linkage disequilibrium between a single nucleotide polymorphism (SNP) and a quantitative trait locus (QTL). When individuals originate from different populations, as in the crossbreeding context, these assumptions are violated because allele frequencies and the linkage disequilibrium patterns across the genome differ between breeds [21–23]. Models that account for breed-specific allele frequencies were tested with simulated and real data and showed no improvement in prediction accuracies [24–26]. Models that, in addition to including breed-specific allele frequencies, also account for breed-specific SNP effects did outperform models in which SNP effects were assumed to be the same across breeds. However, these results were only observed in simulation studies under some conditions (i.e., low SNP density, large training data size, and low breed relatedness) and where breed-of-origin of alleles was assumed to be known without error [15, 27]. With real data from a two-way crossbreeding scheme, Xiang et al. [28] and Lopes et al. [29] reached different conclusions. When using a model that accounted for breed-specific SNP effects compared to a model in which SNP effects were assumed to be the same across breeds, Xiang et al. [28] found improved prediction accuracies and reduced bias of prediction, whereas, Lopes et al. [29] found similar prediction accuracies between the two models. The benefit of a two-way CB is that tracing the breed-of-origin of alleles is relatively straightforward. However, many crossbreeding schemes are based on a three-way cross, for which tracing the breed-of-origin of alleles is considerably more complicated [30]. Recently we have developed a procedure that enables breed-of-origin assignment (BOA) of alleles in three-way CB animals [31]. BOA allows empirical testing of the model that accounts for breed-specific SNP effects in real data. Therefore, the objectives of this study were to evaluate the estimates of variance components and the accuracy of a model that accounts for breed-specific SNP effects using information from both PB and three-way CB pigs for average daily gain (ADG), back fat thickness (BF), and loin depth (LD).

## Methods

### Data

The pig data consisted of three PB populations: Synthetic boar (S), Landrace (LR), and Large White (LW), and a three-way CB population: S (LR × LW) or S (LW × LR), produced by crossing the above-mentioned PB populations. The numbers of available genotypes and phenotypes per trait and per population are in Table 1. All pigs were genotyped using one of the three following SNP panels: Illumina PorcineSNP60.v2 BeadChip (60 K.v2), Illumina PorcineSNP60 BeadChip (60 K), or Illumina PorcineSNP10 BeadChip (10 K). Pigs genotyped with the 60 K or 10 K chips were imputed to the 60 K.v2 panel using FImpute Version 2.2 software [32]. SNP quality control and imputation were applied on the same dataset in a previous study [31], in which more details are provided. The final SNP set for subsequent analyses consisted of 52,164 SNPs. Phenotypes for ADG (g/day), BF (mm), and LD (mm), were measured for most of the PB and CB pigs. ADG for PB was calculated as the difference of on-test body weight measured on average at 60 days of age and off-test body weight measured on average at 173 days of age. ADG for CB was calculated as the difference of on-test body weight measured on average at 70 days of age and body weight at the end of the finishing period, which was on average 120 kg. BF and LD for PB were measured on average at 173 days of age using an ultrasound instrument, while BF and LD for CB were measured on the carcass after slaughter using a probe, named “capteur gras maigre” (CGM; Sydel, France). For all phenotyped pigs, four generations of pedigree information were included.

**Table 1:** Number of genotypes and phenotypes available for each trait and population

Population	Genotypes	ADG	BF	LD
S	2733	2575	2616	2595
LR	4148	2333	3605	2386
LW	7103	5294	6769	5469
CB	1706	1675	1676	1681
Total	15,690	11,877	14,666	12,131

*S* Synthetic boar, *LR* Landrace, *LW* Large White, *CB* three-way crossbred pigs

*ADG* average daily gain, *BF* back fat thickness, *LD* loin depth

### Analyses

#### GBLUP model with breed-specific partial relationship matrices (BOA model)

To account for the breed-specific effect of SNPs, the following 4-trait animal model with three breed-specific partial relationship matrices (${{\bf G}}^{{\left( {{\bf S}} \right)}}$, ${{\bf G}}^{{\left( {{{\bf LR}}} \right)}}$ and ${{\bf G}}^{{\left( {{{\bf LW}}} \right)}}$ was fitted (BOA model):

$${{\bf y}}_{{{\bf S}}} = {{\bf X}}_{{{\bf S}}} {{\bf b}}_{{{\bf S}}} + {{\bf W}}_{{{\bf S}}} {{\bf u}}_{{{\bf S}}} + {{\bf Z}}_{{{\bf S}}} {{\bf a}}_{{{\bf S}}} + {{\bf e}}_{{{\bf S}}} ,$$

$${{\bf y}}_{{{{\bf LR}}}} = {{\bf X}}_{{{{\bf LR}}}} {{\bf b}}_{{{{\bf LR}}}} + {{\bf W}}_{{{{\bf LR}}}} {{\bf u}}_{{{{\bf LR}}}} + {{\bf Z}}_{{{{\bf LR}}}} {{\bf a}}_{{{{\bf LR}}}} + {{\bf e}}_{{{{\bf LR}}}} ,$$

$${{\bf y}}_{{{{\bf LW}}}} = {{\bf X}}_{{{{\bf LW}}}} {{\bf b}}_{{{{\bf LW}}}} + {{\bf W}}_{{{{\bf LW}}}} {{\bf u}}_{{{{\bf LW}}}} + {{\bf Z}}_{{{{\bf LW}}}} {{\bf a}}_{{{{\bf LW}}}} + {{\bf e}}_{{{{\bf LW}}}} ,$$

$${{\bf y}}_{{{{\bf CB}}}} = {{\bf X}}_{{{{\bf CB}}}} {{\bf b}}_{{{{\bf CB}}}} + {{\bf W}}_{{{{\bf CB}}}} {{\bf u}}_{{{{\bf CB}}}} + {{\bf Z}}_{{{{\bf CB}}}} {{\bf g}}_{{{{\bf CB}}}}^{{\left( {{\bf S}} \right)}} + {{\bf Z}}_{{{{\bf CB}}}} {{\bf g}}_{{{{\bf CB}}}}^{{\left( {{{\bf LR}}} \right)}} + {{\bf Z}}_{{{{\bf CB}}}} {{\bf g}}_{{{{\bf CB}}}}^{{\left( {{{\bf LW}}} \right)}} + {{\bf e}}_{{{{\bf CB}}}} ,$$

where ${{\bf y}}_{{{\bf S}}}$, ${{\bf y}}_{{{{\bf LR}}}}$, ${{\bf y}}_{{{{\bf LW}}}}$, and ${{\bf y}}_{{{{\bf CB}}}}$ are the vectors of the phenotypes for S, LR, LW, and CB pigs, respectively; ${{\bf b}}_{{{\bf S}}}$, ${{\bf b}}_{{{{\bf LR}}}}$, ${{\bf b}}_{{{{\bf LW}}}}$, and ${{\bf b}}_{{{{\bf CB}}}}$ represent the vectors of fixed effects (listed in Table 2) and ${{\bf X}}_{{{\bf S}}}$, ${{\bf X}}_{{{{\bf LR}}}}$, ${{\bf X}}_{{{{\bf LW}}}}$, and ${{\bf X}}_{{{{\bf CB}}}}$ are the respective incidence matrices relating pig records to fixed effects; ${{\bf u}}_{{{\bf S}}}$, ${{\bf u}}_{{{{\bf LR}}}}$, ${{\bf u}}_{{{{\bf LW}}}}$, and ${{\bf u}}_{{{{\bf CB}}}}$ represent the vectors of random common litter effects, and ${{\bf W}}_{{{\bf S}}}$, ${{\bf W}}_{{{{\bf LR}}}}$, ${{\bf W}}_{{{{\bf LW}}}}$, and ${{\bf W}}_{{{{\bf CB}}}}$ are the respective incidence matrices relating pig records to litter effects; ${{\bf a}}_{{{\bf S}}}$, ${{\bf a}}_{{{{\bf LR}}}}$, and ${{\bf a}}_{{{{\bf LW}}}}$, are the vectors of additive genetic effects in PB, ${{\bf g}}_{{{{\bf CB}}}}^{{\left( {{\bf S}} \right)}}$, ${{\bf g}}_{{{{\bf CB}}}}^{{\left( {{{\bf LR}}} \right)}}$, and ${{\bf g}}_{{{{\bf CB}}}}^{{\left( {{{\bf LW}}} \right)}}$ are the vectors of the additive genetic effect of PB gametes in CB, and ${{\bf Z}}_{{{\bf S}}}$, ${{\bf Z}}_{{{{\bf LR}}}}$, ${{\bf Z}}_{{{{\bf LW}}}}$, and ${{\bf Z}}_{{{{\bf CB}}}}$ are the respective incidence matrices. Because each model was run for each trait and only pigs with phenotypes were included, ${{\bf Z}}$ incidence matrices relating pig records to additive genetic effects were identity matrices when variance components were estimated. Finally, ${{\bf e}}_{{{\bf S}}}$, ${{\bf e}}_{{{{\bf LR}}}}$, ${{\bf e}}_{{{{\bf LW}}}}$, and ${{\bf e}}_{{{{\bf CB}}}}$ represent the vectors of random residual effects. The variance–covariance of the common litter effect and residual effect were:

$${\text{Var}}\left[ {\begin{array}{*{20}c} {{{\bf u}}_{{{\bf S}}} } \\ {\begin{array}{*{20}c} {{{\bf u}}_{{{{\bf LR}}}} } \\ {{{\bf u}}_{{{{\bf LW}}}} } \\ \end{array} } \\ {{{\bf u}}_{{{{\bf CB}}}} } \\ \end{array} } \right] = \left[ {\begin{array}{*{20}c} {\upsigma_{{u_{S} }}^{2} } & 0 & 0 & 0 \\ 0 & {\upsigma_{{u_{LR} }}^{2} } & 0 & 0 \\ 0 & 0 & {\upsigma_{{u_{LW} }}^{2} } & 0 \\ 0 & 0 & 0 & {\upsigma_{{u_{CB} }}^{2} } \\ \end{array} } \right] \otimes {{\bf I}},$$

and

$${\text{Var}}\left[ {\begin{array}{*{20}c} {{{\bf e}}_{{{\bf S}}} } \\ {\begin{array}{*{20}c} {{{\bf e}}_{{{{\bf LR}}}} } \\ {{{\bf e}}_{{{{\bf LW}}}} } \\ \end{array} } \\ {{{\bf e}}_{{{{\bf CB}}}} } \\ \end{array} } \right] = \left[ {\begin{array}{*{20}c} {\upsigma_{{e_{S} }}^{2} } & 0 & 0 & 0 \\ 0 & {\upsigma_{{e_{LR} }}^{2} } & 0 & 0 \\ 0 & 0 & {\upsigma_{{e_{LW} }}^{2} } & 0 \\ 0 & 0 & 0 & {\upsigma_{{e_{CB} }}^{2} } \\ \end{array} } \right] \otimes {{\bf I}}.$$

**Table 2:** Fixed effects used in the GBLUP models for average daily gain (ADG), back fat thickness (BF), and loin depth (LD), for purebred (PB) (i.e. S, LR, LW) and three-way crossbred (CB) pigs

Trait	Population	Fixed effects
ADG	PB	${\text{farm}}\_{\text{breed}}\_{\text{sex}}\; + \;{\text{b}}_{{\text{a}}} \times {\text{birth}}\;{\text{weight}}$
	CB	${\text{trial}}\; + \;{\text{farm}}\_{\text{sex}}\; + \;{\text{b}}_{{\text{a}}} \; \times \;{\text{birth}}\;{\text{weight}}$
BF, LD	PB	${\text{farm}}\_{\text{breed}}\_{\text{sex}} + {\text{b}}_{{\text{b}}} \times {\text{off\_test}}\;{\text{BW}}$
	CB	${\text{trial}}\; + \;{\text{farm}}\_{\text{sex}} + {\text{b}}_{{\text{c}}} \times {\text{hot}}\;{\text{carcass}}\;{\text{weight}}$

b_a_, b_b_, b_c_, are regression coefficients for birth weight, off-test BW, and hot carcass weight, respectively

The variance–covariance of additive genetic effect for breed S origin was:

$${\text{Var}}\left[ {\begin{array}{*{20}c} {{{\bf a}}_{{{\bf S}}} } \\ {\begin{array}{*{20}c} {{{\bf a}}_{{{{\bf CB}}}}^{{\left( {{\bf S}} \right)}} } \\ {{{\bf g}}_{{{\bf S}}} } \\ \end{array} } \\ {{{\bf g}}_{{{{\bf CB}}}}^{{\left( {{\bf S}} \right)}} } \\ \end{array} } \right] = \left[ {\begin{array}{*{20}c} {\upsigma_{{a_{S} }}^{2} } & {\upsigma_{{a_{S} ,g_{S} }} } \\ {\upsigma_{{g_{S} ,a_{S} }} } & {\upsigma_{{g_{S} }}^{2} } \\ \end{array} } \right] \otimes {{\bf G}}^{{\left( {{\bf S}} \right)}} = \left[ {\begin{array}{*{20}c} {\upsigma_{{a_{S} }}^{2} } & {\upsigma_{{a_{S} ,g_{S} }} } \\ {\upsigma_{{g_{S} ,a_{S} }} } & {\upsigma_{{g_{S} }}^{2} } \\ \end{array} } \right] \otimes \left[ {\begin{array}{*{20}c} {{{\bf G}}_{{{{\bf S}},{{\bf S}}}} } & {{{\bf G}}_{{{{\bf S}},{{\bf CB}}}}^{{\left( {{\bf S}} \right)}} } \\ {{{\bf G}}_{{{{\bf CB}},{{\bf S}}}}^{{\left( {{\bf S}} \right)}} } & {{{\bf G}}_{{{{\bf CB}},{{\bf CB}}}}^{{\left( {{\bf S}} \right)}} } \\ \end{array} } \right],$$

where S pigs have additive effects (i.e. breeding values), ${{\bf a}}_{{{\bf S}}}$ for PB performance and ${{\bf a}}_{{{{\bf CB}}}}^{{\left( {{\bf S}} \right)}}$ for CB performance. The CB pigs have additive effects from the breed S gametes, ${{\bf g}}_{{{{\bf CB}}}}^{{\left( {{\bf S}} \right)}}$ for CB performance and ${{\bf g}}_{{{\bf S}}}$ for PB performance. This last effect, ${{\bf g}}_{{{\bf S}}}$, is an artificial random vector that is added to be able to define the variance–covariance of additive genetic effects with the above Kronecker product, but does not have practical relevance. The matrix ${{\bf G}}^{{\left( {{\bf S}} \right)}}$ is a breed-specific partial relationships matrix for breed S which contains four blocks, one for within S pigs (${{\bf G}}_{{{{\bf S}},{{\bf S}}}}$), two for S with CB pigs (${{\bf G}}_{{{{\bf S}},{{\bf CB}}}}^{{\left( {{\bf S}} \right)}}$ and ${{\bf G}}_{{{{\bf CB}},{{\bf S}}}}^{{\left( {{\bf S}} \right)}}$), and one for within CB pigs (${{\bf G}}_{{{{\bf CB}},{{\bf CB}}}}^{{\left( {{\bf S}} \right)}}$).

The variance–covariance structures for the origin of breeds LR and LW are defined similarly, and the three variance–covariance structures are assumed independent, i.e. no covariances are considered between S, LR, and LW effects [18]. There are six genetic variance components, two for each breed-of-origin, and three covariance components, one for each breed-of-origin. To construct the three breed-specific partial relationship matrices, ${{\bf G}}^{{\left( {{\bf S}} \right)}}$, ${{\bf G}}^{{\left( {{{\bf LR}}} \right)}}$, and ${{\bf G}}^{{\left( {{{\bf LW}}} \right)}}$, we used the breed-of-origin of phased alleles in CB pigs. Then, the breed-specific partial relationship submatrices are defined as, e.g. breed S origin:

$${{\bf G}}_{{{{\bf S}},{{\bf S}}}} = \left( {{{\bf M}}^{{{\bf S}}} - 2{{\bf 1p}}^{{{{\bf S}}^{\prime} }} } \right){{\bf D}}^{{{\bf S}}} \left( {{{\bf M}}^{{{\bf S}}} - 2{{\bf 1p}}^{{{{\bf S}}^{\prime} }} } \right)^{\prime } /N,$$

$${{\bf G}}_{{{{\bf S}},{{\bf CB}}}} = \left( {{{\bf M}}^{{{\bf S}}} - 2{{\bf 1p}}^{{{{\bf S}}^{\prime} }} } \right){{\bf D}}^{{{\bf S}}} \left( {{{\bf M}}^{{{{\bf CB}}}} - {{\bf 1p}}^{{{{\bf S}}^{\prime} }} } \right)^{\prime } /N,$$

$${{\bf G}}_{{{{\bf CB}},{{\bf CB}}}} = \left( {{{\bf M}}^{{{{\bf CB}}}} - {{\bf 1p}}^{{{{\bf S}}^{\prime} }} } \right){{\bf D}}^{{{\bf S}}} \left( {{{\bf M}}^{{{{\bf CB}}}} - {{\bf 1p}}^{{{{\bf S}}^{\prime} }} } \right)^{\prime } /N,$$

where $${{\bf M}}^{{{\bf S}}}$$ is a matrix containing breed-specific allele content for breed S pigs (coded as 0, 1, or 2), $${{\bf M}}^{{{{\bf CB}}}}$$ is a matrix containing breed-specific allele content for CB pigs (coded as 0, or 1), alleles that were not assigned a breed-of-origin were set to missing, $${{\bf p}}^{{{\bf S}}}$$ is the vector of breed S specific frequencies of the counted allele ($$p_{j}^{s} )$$. $$p_{j}^{s}$$ was calculated across S and CB pigs by counting the occurrences of alleles originating from the S breed and coded as 1, across the S breed and in CB, divided by the total number of S alleles in the S breed and CB on locus *j*. $${{\bf D}}^{{{\bf S}}}$$ is diagonal with $$D_{jj}^{S} = \frac{1}{{2p_{j}^{S} \left( {1 - p_{j}^{S} } \right)}}$$. $$N$$ is the number of SNPs.

The breed-specific partial relationship submatrices ${{\bf G}}^{{\left( {{{\bf LR}}} \right)}}$ and ${{\bf G}}^{{\left( {{{\bf LW}}} \right)}}$ are defined similarly to ${{\bf G}}^{{\left( {{\bf S}} \right)}}$. However, the entries of the ${{\bf M}}^{{{{\bf CB}}}}$ matrix containing the breed-specific allele content for CB pigs are set to a missing value if the origin of the allele corresponds to the other maternal line, and effectively does not contribute to the breed-specific partial relationship matrix.

#### Assigning breed-of-origin to alleles in crossbreds

To infer the breed-of-origin of the alleles in CB pigs, we used the BOA approach that was developed by Vandenplas et al. [31]. It consists of three steps: (1) phasing the haplotypes of both PB and CB pigs with AlphaPhase1.1 software [33], (2) determining the unique haplotypes among the PB, and (3) assigning the breed-of-origin for each allele carried on the haplotypes of CB. This approach was applied to the same dataset in a previous study [31]. On average, 95.2% of the alleles of the three-way CB pigs were assigned a breed-of-origin. These alleles with their assigned breed-of-origin were used to build the breed-specific partial relationship matrices. Alleles that were not assigned a breed-of-origin were set to missing, and effectively did not contribute to any of the breed-specific partial relationship matrices.

#### GBLUP model with the genomic relationship matrix

For comparison to the BOA model, the following 4-trait animal model was fitted (${{\bf G}}$ model):

$${{\bf y}}_{{{\bf S}}} = {{\bf X}}_{{{\bf S}}} {{\bf b}}_{{{\bf S}}} + {{\bf W}}_{{{\bf S}}} {{\bf u}}_{{{\bf S}}} + {{\bf Z}}_{{{\bf S}}} {{\bf a}}_{{{\bf S}}} + {{\bf e}}_{{{\bf S}}} ,$$

$${{\bf y}}_{{{{\bf LR}}}} = {{\bf X}}_{{{{\bf LR}}}} {{\bf b}}_{{{{\bf LR}}}} + {{\bf W}}_{{{{\bf LR}}}} {{\bf u}}_{{{{\bf LR}}}} + {{\bf Z}}_{{{{\bf LR}}}} {{\bf a}}_{{{{\bf LR}}}} + {{\bf e}}_{{{{\bf LR}}}} ,$$

$${{\bf y}}_{{{{\bf LW}}}} = {{\bf X}}_{{{{\bf LW}}}} {{\bf b}}_{{{{\bf LW}}}} + {{\bf W}}_{{{{\bf LW}}}} {{\bf u}}_{{{{\bf LW}}}} + {{\bf Z}}_{{{{\bf LW}}}} {{\bf a}}_{{{{\bf LW}}}} + {{\bf e}}_{{{{\bf LW}}}} ,$$

$${{\bf y}}_{{{{\bf CB}}}} = {{\bf X}}_{{{{\bf CB}}}} {{\bf b}}_{{{{\bf CB}}}} + {{\bf W}}_{{{{\bf CB}}}} {{\bf u}}_{{{{\bf CB}}}} + {{\bf Z}}_{{{{\bf CB}}}} {{\bf a}}_{{{{\bf CB}}}} + {{\bf e}}_{{{{\bf CB}}}} ,$$

where vectors and matrices are defined as in the BOA model, with the only difference being that the additive genetic effect in CB pigs was defined only by one vector, ${{\bf a}}_{{{{\bf CB}}}}$. Therefore, the variance–covariance matrix of genetic effects was:

$${\text{Var}}\left[ {\begin{array}{*{20}c} {{{\bf a}}_{{{\bf S}}} } \\ {\begin{array}{*{20}c} {{{\bf a}}_{{{{\bf LR}}}} } \\ {{{\bf a}}_{{{{\bf LW}}}} } \\ \end{array} } \\ {{{\bf a}}_{{{{\bf CB}}}} } \\ \end{array} } \right] = \left[ {\begin{array}{*{20}c} {\upsigma_{{a_{S} }}^{2} } & {\upsigma_{{a_{S} ,a_{LR} }} } & {\upsigma_{{a_{S} ,a_{LW} }} } & {\upsigma_{{a_{S} ,a_{CB} }} } \\ {\upsigma_{{a_{S} ,a_{LR} }} } & {\upsigma_{{a_{LR} }}^{2} } & {\upsigma_{{a_{LR} ,a_{LW} }} } & {\upsigma_{{a_{LR} ,a_{CB} }} } \\ {\upsigma_{{a_{S} ,a_{LW} }} } & {\upsigma_{{a_{LR} ,a_{LW} }} } & {\upsigma_{{a_{LW} }}^{2} } & {\upsigma_{{a_{LW} ,a_{CB} }} } \\ {\upsigma_{{a_{S} ,a_{CB} }} } & {\upsigma_{{a_{LR} ,a_{CB} }} } & {\upsigma_{{a_{LW} ,a_{CB} }} } & {\upsigma_{{a_{CB} }}^{2} } \\ \end{array} } \right] \otimes {{\bf G}}.$$

This model was implemented using two different genomic relationship matrices (${{\bf G}}$) as explained in the next sections.

#### Genomic relationship matrix using allele frequencies across all genotyped pigs (${{\bf G}}_{{{\bf A}}}$ matrix)

The ${{\bf G}}_{{{\bf A}}}$ matrix was constructed using the second method in VanRaden [20]:

$${{\bf G}}_{{{\bf A}}} = \left( {{{\bf M}} - 2{{\bf 1p}}^{^{\prime} } } \right){{\bf D}}\left( {{{\bf M}} - 2{{\bf 1p}}^{\prime } } \right)^{\prime } /N,$$

where $${{\bf M}}$$ is a matrix containing SNP genotypes for each pig (coded as 0, 1, or 2), $${{\bf p}}$$ is the vector of the frequencies of the counted allele ($$p_{j} )$$, calculated across the genotyped population, $${{\bf D}}$$ is diagonal with $$D_{jj} = \frac{1}{{p_{j} \left( {1 - p_{j} } \right)}}$$, and $$N$$ is the number of SNPs.

#### Genomic relationship matrix using breed-specific allele frequencies (${{\bf G}}_{{{\bf B}}}$ matrix)

To account for population structure, we also used a genomic relationship matrix based on genotypes centered and scaled by breed-specific allele frequencies (${{\bf G}}_{{{\bf B}}}$):

$${{\bf G}}_{{{\bf B}}} = \left( {{{\bf M}} - 2{{\bf 1p}}_{{{\bf B}}}^{\prime } } \right){{\bf D}}^{{{\bf B}}} \left( {{{\bf M}} - 2{{\bf 1p}}_{{{\bf B}}} ^{\prime} } \right)^{\prime } /N,$$

where each $${{\bf p}}_{{{\bf B}}}$$ is the vector of the frequencies of the counted allele ($$p_{Bj} )$$. $$p_{Bj}$$ was obtained by summing the contribution of each pure breed $$j$$ and the weighted contribution of the CB. The weight was 0.5 for S, and 0.25 for LR and LW. $${{\bf D}}^{{{\bf B}}}$$ is diagonal with $$D_{jj}^{B} = \frac{1}{{p_{{}} \left( {1 - p_{Bj} } \right)}}$$.

### Estimation of variance components and BLUP

Implementation of the aforementioned GBLUP models required estimates for all variance components involved. Variance components were estimated for each of the three models using the ASReml software [34]. Instead of one 4-trait multivariate model, three bivariate models were fitted to overcome workspace memory limitation of the software. Each analysis included PB of one of the three breeds and all CB. As a consequence, genetic co-variances between breeds were not estimated. For the BOA model, these genetic co-variances are not considered and thus are effectively equal to 0. For the other two models, we also assumed that these co-variances were not significant, and therefore, we set them to 0 in the subsequent BLUP analyses. Variance components of the bivariate models were combined to obtain the full variance–covariance matrices for the 4-trait model. The variance–covariance matrices were combined by averaging the three CB variance components estimated in each of the bivariate models. If necessary, the combined variance–covariance matrices were bended to make them positive definite [35]. Bending changed the variance–covariance components on average by 7.5% (0.3 to 18.5%). BLUP for the three models were obtained using the MiXBLUP software [36].

### Cross-validation

The accuracy of EBV of PB pigs for CB performance from the three models was evaluated as the average accuracy obtained from fourfold cross-validation. Because of different degrees of relationship between PB and CB, genotyped S, LR, or LW pigs were first divided into four mutually exclusive clusters, using the K-means clustering method applied to a dissimilarity matrix computed from elements of the ${{\bf G}}_{{{\bf A}}}$ matrix [37]. Then, each CB pig was assigned to the PB cluster with the closest relationship based on the ${{\bf G}}_{{{\bf A}}}$ matrix. For the maternal breed LW, the CB pigs were not very evenly distributed across the clusters, with one cluster including most of the CB. Therefore, for this breed, the cluster with the largest number of CB pigs was randomly split into four groups and each of those groups was joined with one of the other clusters.

In each training analysis, the data excluded PB and CB pigs from one fold to train on the remaining three folds to predict EBV for CB performance of the excluded PB pigs (validation set). This resulted in every PB pig having EBV for CB performance that were obtained without using performance of the most closely-related CB pigs for training. Thus, the information coming from the most closely-related CB pigs could be used for validation. The number of pigs in the validation and training sets for each of the folds of the cross-validation and for each trait are in Tables 3, 4 and 5 for S, LR, and LW, respectively.

**Table 3:** Cross-validation strategy for crossbred performance of Synthetic boar (S)

Fold	Training		Validation		
	S	CB	S	CB	CB-extra*
*ADG*					
1	2115	1535	460	140	199
2	2119	1341	456	334	268
3	1895	605	680	1070	297
4	1596	1544	979	131	145
*BF*					
1	2132	1536	484	140	188
2	2144	1344	472	332	246
3	1932	604	684	1072	289
4	1640	1544	976	132	145
*LD*					
1	2128	1541	467	140	200
2	2132	1348	463	333	272
3	1921	605	674	1076	299
4	1604	1549	991	132	145

Numbers of individuals for Synthetic boar (S), three-way crossbred (CB) and extra three-way crossbred pigs (CB-extra) in the training and validation sets per trait, i.e. average daily gain (ADG), back fat thickness (BF), and loin depth (LD)

* Three-way crossbred pigs with only phenotypic information, and no genotyping

**Table 4:** Cross-validation strategy for crossbred performance of Landrace (LR)

Groups	Training		Validation		
	LR	CB	LR	CB	CB-extra*
*ADG*					
1	1584	1564	748	111	456
2	1825	1523	507	152	465
3	1762	1531	570	144	456
4	1825	407	507	1268	471
*BF*					
1	2829	1565	775	111	463
2	2492	1523	1112	153	472
3	3002	1532	602	144	463
4	2489	408	1115	1268	478
*LD*					
1	1631	1570	754	111	463
2	1891	1528	494	153	472
3	1823	1537	562	144	463
4	1810	408	575	1273	478

Numbers of individuals for Landrace (LR), three-way crossbred (CB), and extra three-way crossbred pigs (CB-extra) in the training and validation sets per trait, i.e. average daily gain (ADG), back fat thickness (BF), and loin depth (LD)

* Three-way crossbred pigs with only phenotypic information, no genotyped

**Table 5:** Cross-validation strategy for crossbred performance of Large White (LW)

Groups	Training		Validation		
	LR	CB	LR	CB	CB-extra*
*ADG*					
1	3628	1193	1666	482	468
2	3612	1269	1682	406	468
3	4008	1111	1286	564	468
4	4634	1452	660	223	468
*BF*					
1	4870	1191	1899	485	475
2	4954	1271	1815	405	475
3	4381	1113	2388	563	475
4	6102	1453	667	223	475
*LD*					
1	3759	1196	1710	485	475
2	3678	1275	1791	406	475
3	4162	1114	1307	567	475
4	4808	1458	661	223	475

Numbers of individuals for Large White (LW), three-way crossbred (CB), and extra three-way crossbred pigs (CB-extra) in the training and validation sets per trait, i.e. average daily gain (ADG), back fat thickness (BF), and loin depth (LD)

* Three-way crossbred pigs with only phenotypic information, no genotyped

#### Validation set

The PB pigs cannot have an own performance for CB performance, and also in our data, they do not have large offspring groups, which would allow to compute a phenotype as average offspring performance. Therefore, we calculated deregressed proofs (DRP) for PB pigs within the validation sets to validate the predictions of our models. For this, first we obtained EBV from the ${\text{G}}$ model with a pedigree-based relationship matrix. This resulted in an EBV for CB performance for each PB pig. The EBV were estimated based on performance of the CB pigs assigned to each of the validation folds (Tables 3, 4, and 5 for S, LR, and LW, respectively). Phenotype information was also available for an additional 501 CB pigs (CB-extra) that were not genotyped. These records were used in each of the four validation folds (Tables 3, 4, 5 for S, LR, and LW, respectively). Within each validation fold, the EBV of PB pigs for CB performance were then deregressed according to Calus et al. [38]. The deregression involved removal of all effects of relatives in the same validation set, and correction for regression to the mean, to obtain a more accurate estimate of the expected phenotype. In addition, a weighting factor ($w$) was estimated for each DRP value based on the reliability of the calculated DRP. These $w$ are the effective record contributions [39], and reflect the amount of information in the DRP contributed by the animal itself, correcting for any information of the relatives that contributed to its EBV before deregression.

#### Predictive ability

Accuracies of the BOA and ${\text{G}}$ models were calculated as the weighted correlation between the DRP and the EBV of PB pigs for CB performance, where the weighting factor $w$ was used to account for differences in the amount of available information on relatives to estimate DRP. The standard error (SE) of the correlations were approximated as $(1 - {\text{r}}^{2} )/\sqrt {\text{N}}$, were ${\text{r}}$ is the accuracy of the model, and ${\text{N}}$ is the number of validation animals [40].

## Results

### Genotyped population and relationship matrices

**Fig. 1:**
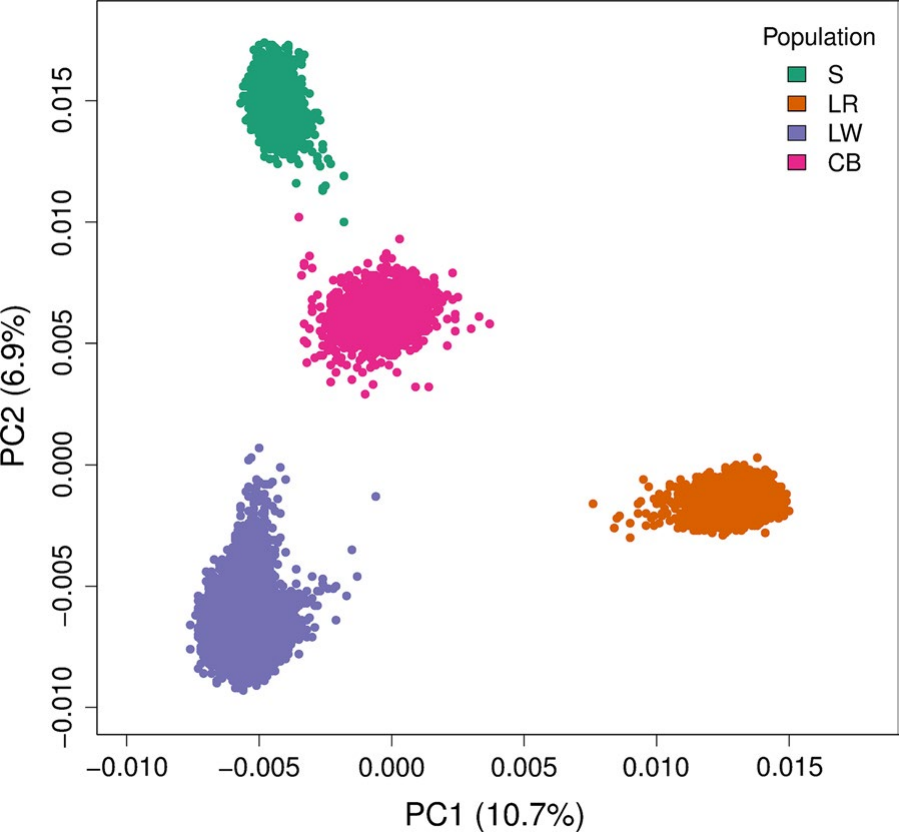
The two first principal components (PC) from the genomic relationship matrix between the different populations. Synthetic boar (S), Landrace (LR), Large White (LW), and three-way crossbred (CB) pigs. Each circle (o) represents a pig

The three breeds, S, LR, and LW, were clearly different populations as shown in Fig. 1 based on the first two principal components of the ${{\bf G}}_{{{\bf A}}}$ matrix. The CB population appeared intermediate among the PB populations. The divergence among the three populations estimated with Weir and Cockerham’s $F_{ST}$ [41], were equal to 0.17 between S and LR, 0.12 between S and LW, and 0.14 between LW and LR, which indicated that they are distantly-related breeds.

The relationships between breeds, calculated with the ${{\bf G}}_{{{\bf A}}}$ matrix were mainly negative (Table 6), with average relationships between breeds ranging from − 0.13 to − 0.07. When using the ${{\bf G}}_{{{\bf B}}}$ matrix, the average relationships between all breeds are zero by definition. When using breed-specific partial relationship matrices (${{\bf G}}^{{\left( {{\bf S}} \right)}}$, ${{\bf G}}^{{\left( {{{\bf LR}}} \right)}}$ and ${{\bf G}}^{{\left( {{{\bf LW}}} \right)}}$), only the relationships based on common alleles originating from the same breed were considered and, consequently no relationships were estimated between breeds. For CB pigs, the diagonal elements of the ${{\bf G}}_{{{\bf A}}}$ and ${{\bf G}}_{{{\bf B}}}$ matrices had an average of 0.96 and 0.94, respectively. For the ${{\bf G}}^{{\left( {{\bf S}} \right)}}$, ${{\bf G}}^{{\left( {{{\bf LR}}} \right)}}$ and ${{\bf G}}^{{\left( {{{\bf LW}}} \right)}}$ matrices, as they are partial relationship matrices, the diagonal elements for CB pigs had averages of 0.49, 0.32, and 0.30 for ${{\bf G}}^{{\left( {{\bf S}} \right)}}$, ${{\bf G}}^{{\left( {{{\bf LR}}} \right)}}$ and ${{\bf G}}^{{\left( {{{\bf LW}}} \right)}}$, respectively. These averages are close to the expected values, i.e. 0.50 for the S breed and 0.25 for the LR and LW breeds.

**Table 6:** Descriptive statistics for relationship between populations based on different genomic relationship matrices

Relationship between	Matrix^a^	Mean	Median	Min	Max	SD
S-LR	${{\bf G}}_{{{\bf A}}}$	− 0.13	− 0.13	− 0.22	0.00	0.02
	${{\bf G}}_{{{\bf B}}}$	0.00	0.00	− 0.09	0.09	0.02
S-LW	${{\bf G}}_{{{\bf A}}}$	− 0.07	− 0.07	− 0.18	0.12	0.02
	${{\bf G}}_{{{\bf B}}}$	0.00	0.00	− 0.11	0.11	0.02
LR-LW	${{\bf G}}_{{{\bf A}}}$	− 0.13	− 0.13	− 0.23	0.16	0.02
	${{\bf G}}_{{{\bf B}}}$	0.00	0.00	− 0.13	0.23	0.02
CB (diagonal)	${{\bf G}}^{{\left( {{\bf S}} \right)}}$	0.49	0.49	0.40	0.80	0.04
	${{\bf G}}^{{\left( {{{\bf LR}}} \right)}}$	0.23	0.23	0.02	0.40	0.04
	${{\bf G}}^{{\left( {{{\bf LW}}} \right)}}$	0.23	0.23	0.07	0.39	0.04
	${{\bf G}}_{{{\bf A}}}$	0.96	0.95	0.88	1.07	0.03
	${{\bf G}}_{{{\bf B}}}$	0.94	0.93	0.86	1.08	0.03

^a^ ${{\bf G}}^{{\left( {{\bf S}} \right)}}$ = partial relationship matrix for breed Synthetic boar (S); ${{\bf G}}^{{\left( {{{\bf LR}}} \right)}}$ = partial relationship matrix for breed Landrace (LR); ${{\bf G}}^{{\left( {{{\bf LW}}} \right)}}$ = partial relationship matrix for breed Large White (LW); ${{\bf G}}_{{{\bf A}}}$ = genomic relationship matrix by allele frequencies obtained across the genotyped population; ${{\bf G}}_{{{\bf B}}}$ = genomic relationship matrix by breed-specific allele frequencies

### Variance components, heritabilities, and genetic correlations

Estimated variance components for ADG, BF, and LD using the BOA model with the ${{\bf G}}^{{\left( {{\bf S}} \right)}}$, ${{\bf G}}^{{\left( {{{\bf LR}}} \right)}}$ and ${{\bf G}}^{{\left( {{{\bf LW}}} \right)}}$ matrices, the ${\text{G}}$ model with the ${{\bf G}}_{{{\bf A}}}$ matrix (${\text{G}}_{\text{A}}$ model), and the ${\text{G}}$ model with ${{\bf G}}_{{{\bf B}}}$ matrix (${\text{G}}_{\text{B}}$ model) are in Table 7. The standard errors of the estimated variance components in Table 7 are provided in Additional file 1: Table S1. Regardless of the model and trait, the PB additive genetic variance estimated for the maternal breeds, i.e. LR and LW, were very similar. For the maternal breeds, CB additive genetic variance was larger than PB additive genetic variance for all traits. For the paternal breed, the opposite was observed, i.e. CB additive genetic variance was smaller than PB additive genetic variance, for all traits except BF. Estimates of CB heritability tended to be higher than estimates of PB heritability for all traits except LD.

**Table 7:** Additive genetic variance ($\varvec{\sigma}_{\varvec{a}}^{2}$), litter variance ($\varvec{\sigma}_{\varvec{u}}^{2}$), residual variance ($\varvec{\sigma}_{\varvec{e}}^{2}$), and heritabilities for each breed for PB and CB performance, and genetic correlation between purebred and CB pigs ($\varvec{r}_{{\varvec{PC}}}$), estimated for each trait using the BOA^a^, G_A_^b^, and G_B_^c^ models

Model	Breed	${\varvec{\upsigma}}_{{{{\bf a}}_{{{{\bf PB}}}} }}^{2}$	${\varvec{\upsigma}}_{{{{\bf u}}_{{{{\bf PB}}}} }}^{2}$	${\varvec{\upsigma}}_{{{{\bf e}}_{{{{\bf PB}}}} }}^{2}$	$\varvec{h}_{{{{\bf PB}}}}^{2}$	${\varvec{\upsigma}}_{{{{\bf a}}_{{{{\bf CB}}}} }}^{2}$	${\varvec{\upsigma}}_{{{{\bf u}}_{{{{\bf CB}}}} }}^{2}$ *	${\varvec{\upsigma}}_{{{{\bf e}}_{{{{\bf CB}}}} }}^{2}$ *	$\varvec{h}_{{{{\bf CB}}}}^{2}$	${{\bf r}}_{{{{\bf pc}}}}$
*ADG*										
BOA	S	2699	2925	6124	0.23	2316	853	4192	0.34**	0.50
	LR	2165	2291	3778	0.26	3566				0.62
	LW	2123	1595	4602	0.26	2258				0.57
G_A_	S	3386	2850	6068	0.28	2053*	258	3576	0.35	0.52
	LR	2461	2282	3718	0.29					0.31
	LW	2336	1563	4595	0.28					0.61
G_B_	S	2775	2846	6082	0.24	2261*	262	3592	0.37	0.52
	LR	2248	2287	3703	0.27					0.30
	LW	2154	1640	4568	0.26					0.59
*BF*										
BOA	S	0.82	0.55	1.27	0.31	1.90	0.88	3.96	0.38**	0.74
	LR	1.09	0.60	1.73	0.32	3.74				0.67
	LW	1.33	0.86	1.67	0.34	4.16				0.58
G_A_	S	1.18	0.55	1.26	0.40	2.18*	0.33	3.32	0.37	0.73
	LR	1.38	0.59	1.71	0.38					0.72
	LW	1.57	0.85	1.64	0.39					0.65
G_B_	S	0.98	0.54	1.26	0.35	2.40*	0.34	3.34	0.39	0.69
	LR	1.26	0.59	1.70	0.35					0.70
	LW	1.44	0.85	1.64	0.37					0.62
*LD*										
BOA	S	10.59	6.00	8.43	0.42	11.59	3.20	31.45	0.24**	0.53
	LR	5.72	3.00	6.65	0.37	7.23				0.58
	LW	6.04	3.55	6.93	0.37	12.86				0.53
G_A_	S	12.78	5.93	8.41	0.47	9.05*	0.11	28.89	0.24	0.57
	LR	6.58	2.98	6.60	0.41					0.57
	LW	6.82	3.56	6.89	0.40					0.68
G_B_	S	10.58	5.87	8.33	0.43	10.00*	0.05	28.89	0.26	0.55
	LR	5.82	2.97	6.57	0.38					0.56
	LW	6.09	3.55	6.86	0.37					0.62

*S* Synthetic boar, *LR* Landrace, *LW* Large White, *CB* three-way crossbred pigs

*ADG* average daily gain, *BF* back fat thickness, *LD* loin depth

^a^BOA model, model with breed-specific relationship matrices

^b^ ${\text{G}}_{\text{A}}$ model, model with genomic relationship matrix by allele frequencies obtained across the genotyped population

^c^ ${\text{G}}_{\text{B}}$ model, model with genomic relationship matrix by breed-specific allele frequencies

* Average from the three bivariate models

** (0.5$$\upsigma_{{{\text{a}}_{\text{S}} }}^{2}$$ + 0.25$$\upsigma_{{{\text{a}}_{\text{LR}} }}^{2}$$ + 0.25$$\upsigma_{{{\text{a}}_{\text{LW}} }}^{2}$$)/(0.5$$\upsigma_{{{\text{a}}_{\text{S}} }}^{2}$$ + 0.25$$\upsigma_{{{\text{a}}_{\text{LR}} }}^{2}$$ + 0.25$$\upsigma_{{{\text{a}}_{\text{LW}} }}^{2}$$ + $$\upsigma_{{{\text{u}}_{\text{CB}} }}^{2}$$* + $$\upsigma_{{{\text{e}}_{\text{CB}} }}^{2}$$*)

A comparison between models showed that PB and CB additive genetic variances for the maternal breeds were similar between the ${\text{G}}_{\text{A}}$ and ${\text{G}}_{\text{B}}$ models. For the paternal breed S, compared to the ${\text{G}}_{\text{B}}$ model, the ${\text{G}}_{\text{A}}$ model estimated a larger PB additive genetic variance, and smaller CB additive genetic variance. Estimated PB additive genetic variances with the BOA model were similar to those obtained with the ${\text{G}}_{\text{A}}$ or ${\text{G}}_{\text{B}}$ models and the estimated CB additive genetic variances with the BOA model, on average across the three breeds, were larger than those obtained with the ${\text{G}}_{\text{A}}$ or ${\text{G}}_{\text{B}}$ models. The estimates of PB and CB heritability were similar across models, while estimates obtained with the BOA model tended to be slightly lower and those with the ${\text{G}}_{\text{B}}$ model tended to be slightly higher than with the ${\text{G}}_{\text{A}}$ model. The genetic correlations for traits between PB and CB pigs estimated with the BOA model were generally similar to those of the ${\text{G}}_{\text{A}}$ and ${\text{G}}_{\text{B}}$ models, except for the genetic correlation between LR and CB pigs for ADG that was much higher than that estimated with the ${\text{G}}_{\text{A}}$ and ${\text{G}}_{\text{B}}$ models. The genetic correlations between PB and CB pigs estimated with the ${\text{G}}_{\text{A}}$ or ${\text{G}}_{\text{B}}$ models were similar. In general, the SE of PB additive genetic variances and heritabilities were similar across models, although the SE of the three CB additive genetic variances estimated with the BOA model were much larger than the SE of the single CB additive genetic variance estimated with the ${\text{G}}_{\text{A}}$ or ${\text{G}}_{\text{B}}$ models. The SE of the estimated genetic correlations were relatively large, ranging from 0.10 to 0.29, across all models and traits.

For the BOA model, the CB variance for litter effect was about three times larger than that obtained with the ${\text{G}}_{\text{A}}$ or ${\text{G}}_{\text{B}}$ models. Estimates of the CB residual variance were also slightly larger when using the BOA model compared to the ${\text{G}}_{\text{A}}$ and ${\text{G}}_{\text{B}}$ models. Estimates of PB variance for litter and residual effects by the ${\text{G}}_{\text{A}}$ and ${\text{G}}_{\text{B}}$ models were similar among breeds. Estimates of CB variance for litter and residual effects by the ${\text{G}}_{\text{A}}$ and ${\text{G}}_{\text{B}}$ models were similar among the maternal breeds, while for breed S, the CB variance for litter and residual effects was lower with the ${\text{G}}_{\text{A}}$ model than with the ${\text{G}}_{\text{B}}$ model. In summary, estimated variance components were mostly similar across models, apart from the CB litter variance that was considerably larger with the BOA model compared to the other two models.

### Predicting breeding values of PB pigs for CB performance with different models

**Fig. 2:**
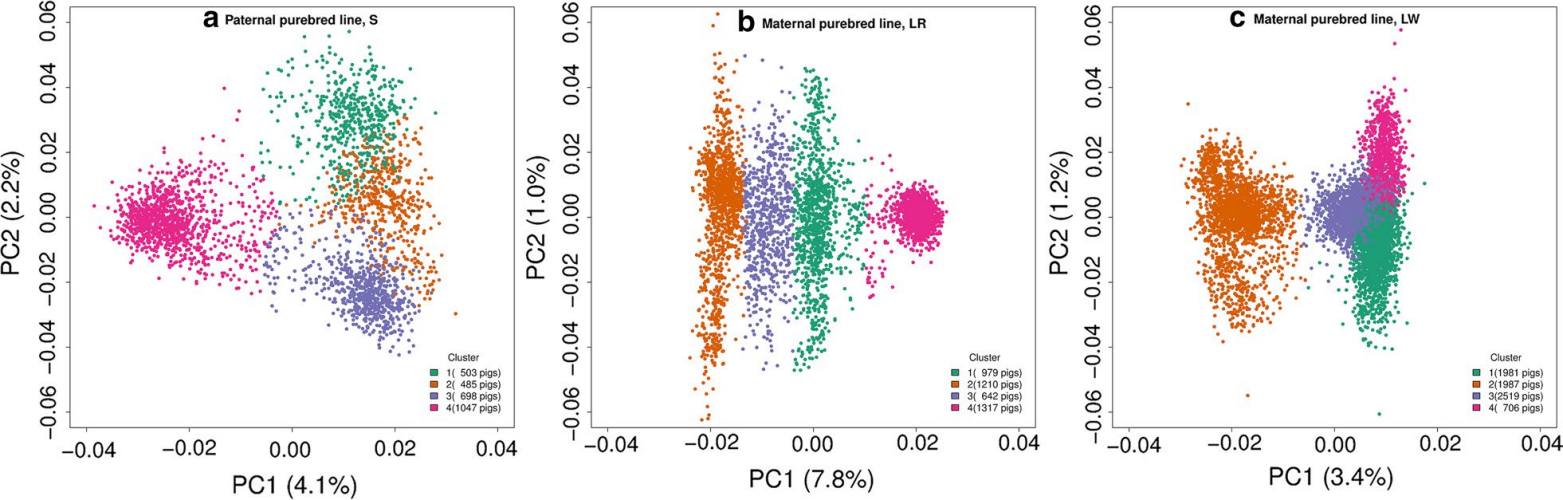
The two first principal components (PC) from the genomic relationship matrix between the four validation groups of Synthetic boar (S) pigs (**a**), Landrace (LR) pigs (**b**) and Large White (LW) pigs (**c**). Each circle (o) represents a pig

For each breed S, LR, and LW, four validation groups were formed to perform the 4-fold cross-validation. Figure 2 represents the first two principal components from the ${{\bf G}}_{{{\bf A}}}$ matrix and shows that the grouping for the cross-validation was done correctly. The first two principal components explained 6.3% of the variability among S pigs, 8.8% among LR pigs and 4.65% among LW pigs.

Accuracies of the three models for the estimated breeding values of S pigs for CB performance are in Table 8. For ADG, the BOA model yielded slightly better accuracies than the ${\text{G}}_{\text{A}}$ and ${\text{G}}_{\text{B}}$ models. The opposite was observed for BF and LD, where the ${\text{G}}_{\text{A}}$ and ${\text{G}}_{\text{B}}$ models yielded slightly better accuracies than the BOA model. Accuracies of the three models for the estimated breeding values of LR pigs for CB performance are in Table 9. For ADG, the BOA model yielded higher accuracies than the ${\text{G}}_{\text{A}}$ and ${\text{G}}_{\text{B}}$ models. For BF and LD, there was no difference in accuracies between the three models. Accuracies of the three models for the estimated breeding values of LW pigs for CB performance are in Table 10. The trait ADG is not included, because the reliabilities of the EBV of LW pigs within the validation groups for CB performance for this trait were too low to be used for proper validation. Similar to the results for the LR breed, there was no difference in accuracies between the three models for the traits BF and LD. In general, accuracies from models ${\text{G}}_{\text{A}}$ and ${\text{G}}_{\text{B}}$ were similar.

**Table 8:** Accuracies* of BOA^a^, G_A_^b^, and G_B_^c^ models calculated for each of the four folds of cross-validation for estimating breeding values of the paternal breed Synthetic boar pigs for crossbred performance for each trait, and average weighting factor (*w*) of the calculated DRP per validation fold

Folds	$\varvec{w}$	BOA	${{\bf G}}_{{{\bf A}}}$	${{\bf G}}_{{{\bf B}}}$
*ADG*				
1	0.49	0.055	0.055	0.057
2	0.12	0.128	0.111	0.094
3	0.21	0.170	0.156	0.152
4	0.07	0.063	0.084	0.082
Mean		0.104	0.102	0.096
*BF*				
1	0.31	0.168	0.168	0.162
2	0.39	0.201	0.157	0.159
3	0.52	0.191	0.294	0.280
4	0.25	0.150	0.179	0.177
Mean		0.178	0.199	0.195
*LD*				
1	0.55	0.204	0.234	0.236
2	0.67	0.212	0.209	0.207
3	0.88	0.127	0.140	0.134
4	0.45	0.088	0.135	0.142
Mean		0.158	0.179	0.180

*ADG* average daily gain, *BF* back fat thickness, *LD* loin depth

* Accuracies measured as weighted correlation between DRP and EBV of S pigs for crossbred performance. Approximate standard errors SE, computed as (1−r^2^)/N √ (1−r^2^)/N, were equal to 0.023 to 0.024 for the mean accuracies across the folds, for all combinations of traits and methods.

^a^BOA model, model with breed-specific relationship matrices

^b^ ${\text{G}}_{\text{A}}$ model, model with genomic relationship matrix by allele frequencies obtained across the genotyped population

^c^ ${{\bf G}}_{{{\bf B}}}$ model, model with genomic relationship matrix by breed-specific allele frequencies

**Table 9:** Accuracies* of BOA^a^, ${{\bf G}}_{{{\bf A}}}$ ^b^, and ${{\bf G}}_{{{\bf B}}}$ ^c^ models calculated for each of the four folds of cross-validation for estimating breeding values of the maternal breed Landrace pigs for crossbred performance for each trait, and weighting factor ($\varvec{w}$) of the calculated DRP

Folds	$\varvec{w}$	BOA	${{\bf G}}_{{{\bf A}}}$	${{\bf G}}_{{{\bf B}}}$
*ADG*				
1	0.20	0.133	0.106	0.099
2	0.23	0.190	0.095	0.111
3	0.21	0.159	0.106	0.106
4	0.22	0.094	0.007	0.014
Mean		0.144	0.079	0.083
*BF*				
1	0.09	0.185	0.169	0.171
2	0.07	0.186	0.210	0.199
3	0.10	0.223	0.216	0.215
4	0.09	0.144	0.149	0.141
Mean		0.184	0.186	0.181
*LD*				
1	0.43	0.224	0.206	0.203
2	0.47	0.085	0.107	0.107
3	0.45	0.239	0.232	0.228
4	0.47	0.170	0.208	0.207
Mean		0.179	0.188	0.186

*ADG* average daily gain, *BF* back fat thickness, *LD* loin depth

* Accuracies measured as weighted correlation between DRP and EBV of LR pigs for crossbred performance. Approximate standard errors SE, computed as (1−r^2^)/N √ (1−r^2^)/N, were equal to 0.023 to 0.024 for the mean accuracies across the folds, for all combinations of traits and methods.

^a^BOA model, model with breed-specific relationship matrices

^b^ ${\text{G}}_{\text{A}}$ model, model with genomic relationship matrix by allele frequencies obtained across the genotyped population

^c^ ${\text{G}}_{\text{B}}$ model, model with genomic relationship matrix by breed-specific allele frequencies

**Table 10:** Accuracies* of BOA^a^, G_A_^b^, and G_B_^c^ models calculated for each of the four folds of cross-validation for estimating breeding values of the maternal breed Large White pigs for crossbred performance for each trait, and weighting factor (w) of the calculated DRP

Folds	$\varvec{w}$	BOA	${{\bf G}}_{{{\bf A}}}$	${{\bf G}}_{{{\bf B}}}$
*BF*				
1	0.21	0.217	0.221	0.216
2	0.13	0.095	0.094	0.089
3	0.28	0.190	0.175	0.170
4	0.23	0.219	0.242	0.243
Mean		0.180	0.183	0.180
*LD*				
1	0.62	0.235	0.234	0.232
2	0.38	0.103	0.126	0.126
3	0.74	0.226	0.229	0.228
4	0.64	0.297	0.318	0.318
Mean		0.215	0.227	0.226

*ADG* average daily gain, *BF* back fat thickness, *LD* loin depth

* Accuracies measured as weighted correlation between DRP and EBV of LR pigs for crossbred performance. Approximate standard errors SE, computed as (1−r^2^)/N √ (1−r^2^)/N, were equal to 0.023 to 0.024 for the mean accuracies across the folds, for all combinations of traits and methods.

^a^BOA model, model with breed-specific relationship matrices

^b^ ${\text{G}}_{\text{A}}$ model, model with genomic relationship matrix by allele frequencies obtained across the genotyped population

^c^ ${\text{G}}_{\text{B}}$ model, model with genomic relationship matrix by breed-specific allele frequencies

## Discussion

### Properties of the relationship matrices

Genomic relationships within and across populations are defined differently depending on how the genetic covariance between individuals is calculated. Using across-breed allele frequencies when the correlations of allele frequencies between breeds differ from 1, could lead to genomic relationships between animals of different breeds that are on average negative [26], as observed for the ${{\bf G}}_{{{\bf A}}}$ matrix. This was not the case for the ${{\bf G}}_{{{\bf B}}}$ matrix, in which the genomic relationships between animals of different breeds was on average 0, as expected for distantly-related breeds.

**Fig. 3:**
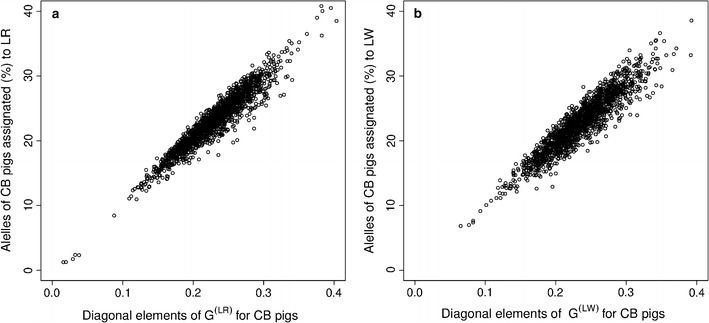
Relation between percentage of assigned alleles to a breed-of-origin and diagonal elements of partial relationship matrices. **a** Observed percentage of assigned alleles of crossbred pigs to Landrace (LR) as breed-of-origin on the y-axis compared to the diagonal elements of the ${{\bf G}}^{{\left( {{{\bf LR}}} \right)}}$ partial relationship matrix for the same crossbred pigs on the x-axis. **b** Observed percentage of assigned alleles of crossbred pigs to Large White (LW) as breed-of-origin on the y-axis compared to the diagonal elements of the ${{\bf G}}^{{\left( {{{\bf LW}}} \right)}}$ partial relationship matrix for the same crossbred pigs on the x-axis

Diagonal elements (${\text{D}}$) from a pedigree-based relationship matrix have a value of 1 when there is no inbreeding. Because a genomic relationship matrix is built to resemble a pedigree-based relationship matrix and the current genotyped population is considered the base population [20], the average ${\text{D}}$ from a genomic relationship matrix is expected to be 1, as we observed for the ${{\bf G}}_{{{\bf A}}}$ and ${{\bf G}}_{{{\bf B}}}$ matrices. To calculate the partial relationship matrices, ${{\bf G}}^{{\left( {{\bf S}} \right)}}$, ${{\bf G}}^{{\left( {{{\bf LR}}} \right)}}$ and ${{\bf G}}^{{\left( {{{\bf LW}}} \right)}}$, the ${\text{D}}$ for CB pigs were expected to be 0.5 for ${{\bf G}}^{{\left( {{\bf S}} \right)}}$, and 0.25 for ${{\bf G}}^{{\left( {{{\bf LR}}} \right)}}$ and ${{\bf G}}^{{\left( {{{\bf LW}}} \right)}}$, expressing the proportion of the genome in CB pigs contributed by each breed S, LR, and LW, respectively. Using all 52,164 SNPs, Fig. 3 shows how the diagonal elements among CB pigs from the ${{\bf G}}^{{\left( {{{\bf LR}}} \right)}}$, and ${{\bf G}}^{{\left( {{{\bf LW}}} \right)}}$ matrices increased as the percentage of alleles of CB pigs assigned to the respective maternal breed as breed-of-origin increased.

### Variance components across models

Estimating variance components for the 4-trait multivariate models was not possible due to workspace memory limitation when trying to run the full BOA model with the three partial relationship matrices or the ${\text{G}}$ models with the relationship matrices containing the four populations. Therefore, for the ${\text{G}}$ models, the construction of a full variance–covariance matrix based on sub-models was required, in this case three bivariate models. This procedure of constructing a full variance–covariance matrix is often used in genetic evaluation [35]. The combined variance–covariance matrices in the ${\text{G}}_{\text{A}}$ and ${\text{G}}_{\text{B}}$ models for BF were considerably bended (variance components changed up to 10.9%) and this may have affected the results. The combined variance–covariance matrix in the ${\text{G}}_{\text{A}}$ model for LD was also bended, but, in this case, the components changed only up to 2.5%. For ADG, no bending of the variance–covariance matrix was required for any of the models. An advantage of the BOA model, since variance–covariance matrices are by breed, is that it allows the estimates of the CB additive genetic variance contributed by the different parental breeds to differ. With the ${\text{G}}_{\text{A}}$ and ${\text{G}}_{\text{B}}$ models, these differences cannot be observed because there is only one estimate for CB additive genetic variance across the three breeds. A disadvantage of the BOA model is that estimates must be based on half the information (for the paternal breed) or on a quarter of the information (for the maternal breeds) compared to estimates from the ${\text{G}}_{\text{A}}$ or ${\text{G}}_{\text{B}}$ models. Therefore, the SE of CB additive genetic variances estimated with the BOA model were much larger than the SE of CB additive genetic variances estimated with the ${\text{G}}_{\text{A}}$ and ${\text{G}}_{\text{B}}$ models. With the BOA model, we could observe that estimates of CB additive genetic variance differed between the three breeds for all traits. This means that ${\text{r}}_{\text{pc}}$ should also be interpreted separately by breed. The estimates of ${\text{r}}_{\text{pc}}$ differed slightly across models. In theory, the CB additive variance components estimated with the BOA model comprises the variation observed in CB pigs due only to the alleles coming from the analyzed breed. Therefore, differences in ${\text{r}}_{\text{pc}}$ estimated with the ${\text{G}}_{\text{A}}$ or ${\text{G}}_{\text{B}}$ model rather than the BOA model were expected. For instance, for ADG, the ${\text{r}}_{\text{pc}}$ estimated with the BOA model for S and LW were slightly smaller than those estimated with the ${\text{G}}_{\text{A}}$ and ${\text{G}}_{\text{B}}$ models. However, the ${\text{r}}_{\text{pc}}$ estimated with the BOA model for LR was twice as high compared to that of the other two models. One explanation is that a large part of the CB additive variance can come mainly from variation observed among the alleles originating from a specific breed and this is not captured when all alleles are assumed to have the same origin.

In the literature, ${\text{r}}_{\text{pc}}$ for production traits have been calculated from pedigree information only [6, 42] and vary greatly, but on average they are higher than our estimates, probably because the breeds were different or the estimates were an average across different breeds. In general, the investigated traits showed a moderate ${\text{r}}_{\text{pc}}$ indicating that using CB information together with PB information in the reference population might be beneficial for selection of PB pigs for CB performance. Using CB information is expected to be most important for combinations of trait and breed for which ${\text{r}}_{\text{pc}}$ is low, for instance for ADG in breed LR.

From the estimates of the BOA model, we observed that CB litter effect and residual variance were much larger than those obtained with the ${\text{G}}_{\text{A}}$ or ${\text{G}}_{\text{B}}$ models. Because the genotypes of only one breed at a time were used in the bivariate BOA model, the litter and residual effect variance in the BOA model likely absorbed the variance coming from the genetic relationships from the breeds that were absent in the model. To investigate the impact of these possibly inflated litter and residual variances, we tried to correct this by setting the CB litter effect and residual variance of the BOA model equal to the average estimates from the ${\text{G}}_{\text{A}}$ and ${\text{G}}_{\text{B}}$ models. Using these new variance estimates did not affect the accuracies of the BOA model compared to the ${\text{G}}_{\text{A}}$ and ${\text{G}}_{\text{B}}$ models (results not shown).

### Predictive ability across models

The three breeds used in this study are distantly related and correlations between breed-specific allele frequencies were low: 0.31 for breeds S and LR, 0.54 for breeds S and LW, and 0.39 for breeds LR and LW. However, taking population structure into account by accounting for different allele frequencies in the three different breeds (${\text{G}}_{\text{B}}$ model) did not improve the accuracy for predicting EBV compared with using allele frequencies obtained across genotyped populations (${\text{G}}_{\text{A}}$ model). In a study with CB sheep, Moghaddar et al. [25] reported limited impact on prediction accuracy when adjusting for breed-specific allele frequency, also when differences in allele frequencies between breeds were large. Makgahlela et al. [24] and Lourenco et al. [26] also observed no advantage of using breed-specific allele frequencies for constructing the relationship matrix, even when this led to observable changes in the coefficients of the relationship matrix. Although correlations between breed-specific allele frequencies were low, correlations between these breed-specific allele frequencies and the across-breed frequency were relatively high, simply because the breed-specific allele frequencies are included in the across-breed allele frequency. In our study, the correlations between the breed-specific allele frequencies and the across-breed frequency were equal to 0.74, 0.68, and 0.89, for breeds S, LR, and LW, respectively. The correlation between breed LW allele frequency and the across-breed frequency was higher than the others, because the LW breed has the largest number of pigs (Table 1), therefore, it has a larger contribution to the across-breed allele frequencies across breeds. The correlation between crossbred allele frequency and the across-breed frequency was equal to 0.93. Therefore, using breed-specific or across-breed frequencies in the calculation of the relationship coefficient between a PB and CB pig will have little effect on predicted EBV of PB for CB performance.

In the ${\text{G}}_{\text{A}}$ and ${\text{G}}_{\text{B}}$ models, genetic co-variances between breeds were assumed to be zero. To test if this was a correct assumption, covariances between PB lines were also estimated by fitting three additional bivariate models (one for each pair of PB) for the trait ADG using the ${\text{G}}_{\text{A}}$ model. Variance components of the six bivariate models were combined to obtain the full variance–covariance matrices for the 4-trait model. This combination was performed by averaging the three variance components estimated for each population, i.e. S, LR, LW and CB. In this case, it was not necessary to bend the combined variance–covariance matrix to make it positive definite. The genetic correlations between PB performance for ADG were 0.13 (± 0.24) between S and LR, 0.39 (± 0.14) between S and LW, and 0.36 (± 0.16) between LR and LW. These estimates were in line with estimated values of 0.23 and 0.30 between a Danish Landrace and Danish Yorkshire population [43]. Moreover, for breeds S and LR, the value of zero was within one SD. Accuracies of the ${\text{G}}_{\text{A}}$ model taking into account the covariance between PB for estimating breeding values of S pigs for CB performance, were similar to prediction accuracy of the ${\text{G}}_{\text{A}}$ model assuming the covariances between PB to be zero (Table 11). This was expected because relationships between pigs from different breeds were low and showed very little variation (Table 6). Therefore, the ${\text{G}}_{\text{A}}$ model assuming the covariances between PB to be zero are not expected to affect accuracies, even when genetic correlations between PB are moderate.

**Table 11:** Accuracies* of the ${{\bf G}}_{{{\bf A}}}$ ^a^ model assuming zero covariance between purebreds and with covariances calculated between purebreds (${{\bf G}}_{{{{\bf A}} - {{\bf covariancePB}}}}$), for each of the four folds of cross-validation for estimating breeding values of the paternal breed Synthetic boar pigs for crossbred performance for average daily gain (ADG)

Folds	${{\bf G}}_{{{\bf A}}}$ ^a^	${{\bf G}}_{{{{\bf A}} - {{\bf covariancePB}}}}$
1	0.055	0.054
2	0.111	0.110
3	0.156	0.171
4	0.084	0.085
Mean	0.102	0.105

* Accuracies measured as weighted correlation between DRP and EBV of S pigs for crossbred performance

^a^ ${\text{G}}_{\text{A}}$ model, model with genomic relationship matrix by allele frequencies obtained across the genotyped population. Results are the same as in Table 8

The BOA model assumes that relationships between PB are zero, and thus also effectively assumes that the covariances between PB are zero. A study from Xiang et al. [43] compares the BOA approach in a single-step model against a single-step model with metafounders, where the last model defines relationships between the pedigree base populations across breeds but also takes genomic relationships across breeds into account. Taken together their conclusions that both models perform similarly and our findings, these results suggest that considering genomic relationships and covariances between PB lines has limited relevance in models for predicting crossbred performance for pig crossbreeding programs.

Compared to the ${\text{G}}_{\text{A}}$ and ${\text{G}}_{\text{B}}$ models, taking population structure into account by using breed-specific partial relationships as in the BOA model, including breed-specific allele frequencies, had some impact on the accuracy of EBV. The BOA model had a positive impact for traits with a low ${\text{r}}_{\text{pc}}$ as for ADG in breed LR (0.30). BF and LD showed higher ${\text{r}}_{\text{pc}}$ (0.55 to 0.73), and accuracies of the BOA model for these traits was similar to those of the ${\text{G}}_{\text{A}}$ or ${\text{G}}_{\text{B}}$ models. Comparing PB lines, somewhat higher accuracies could have been expected for the S line, because the sire line contributes 50% of the genome of the CB, while the dam lines contribute only 25%. Thus, the sire line will have a larger variance in genomic relationships with the CB pigs used for training, which is expected to yield higher accuracies [15]. Nevertheless, in our study, accuracies were very comparable across the sire and dam lines. The BOA model was previously tested on simulated data [15, 27], and on real data but for a two-breed cross scheme [28, 29]. These studies also compared the BOA model to models similar to ${\text{G}}_{\text{A}}$ and ${\text{G}}_{\text{B}}$. Ibánez-Escriche et al. [15] used a simulated population of two-way and three-way CB, for a trait with a heritability of 0.3. They observed that the prediction accuracy of EBV of PB pigs for CB performance with the ${\text{G}}_{\text{A}}$ model was often equal or higher compared to that with the BOA model. The superiority of the BOA model was only observed when PB populations were distant or unrelated, and SNP density was low. Similarly, Esfandyari et al. [27] tested the BOA model with a simulated two-way CB population for a trait with a heritability of 0.3 and a ${\text{r}}_{\text{pc}}$ of 0.78. They observed a higher response to selection in CB animals when the BOA model was used compared to the ${\text{G}}_{\text{A}}$ model, but, again, only when PB populations were distantly related. Vandenplas et al. [44] predicted the average reliability of EBV for CB performance obtained from the ${\text{G}}_{\text{B}}$ and BOA models using simulated PB and two-way CB data and different heritabilities (0.20, 0.40, and 0.95), ${\text{r}}_{\text{pc}}$ (0.30 and 0.70), and population relatedness. In their study, average reliabilities of the BOA model were always lower than those of the ${\text{G}}_{\text{B}}$ model. The difference in reliabilities between the BOA and ${\text{G}}_{\text{B}}$ models also increased with increasing heritability, ${\text{r}}_{\text{pc}}$ and with the population relatedness. Using real data of two-way CB, Xiang et al. [28] and Lopes et al. [29] tested the BOA approach. Xiang et al. [28] used a single-step model with a trait that had a CB heritability of 0.10 and ${\text{r}}_{\text{pc}}$ of 0.59 and 0.73 between each breed. They obtained up to 13% higher accuracy for EBV of PB pigs for CB performance considering breed-specific SNP effects. Lopes et al. [29] tested the BOA approach with two traits that had a CB heritability of 0.14 and 0.37, respectively and ${\text{r}}_{\text{pc}}$ higher than 0.88. They obtained similar prediction accuracies with the BOA approach than with a model that did not account for breed-specific SNP effects in CB animals. The results from these studies indicate that breeding values are better estimated with the BOA model for traits with a low heritability and low ${\text{r}}_{\text{pc}}$. In our study, CB and PB heritabilities were higher than 0.22, which may have limited the positive impact of the BOA model. Therefore, already considering distantly-related breeds, the BOA model seems to outperform the ${\text{G}}_{\text{A}}$ and ${\text{G}}_{\text{B}}$ models for predicting breeding values of PB animals for CB performance, only when the ${\text{r}}_{\text{pc}}$ and heritabilities of the analysed trait are low.

## Conclusions

A positive impact of the BOA model was observed for ADG in breed LR, which showed a low ${\text{r}}_{\text{pc}}$ (0.30). Results from the literature and from our study suggest that, in cases where traits have a combination of low ${\text{r}}_{\text{pc}}$ and low heritabilities, and breeds are distantly related, the use of the BOA model is justified. In other cases, using CB information in a model that does not account for breed-specific SNP effects in CB animals, such as the ${\text{G}}_{\text{A}}$ and ${\text{G}}_{\text{B}}$ models, does not seem to jeopardize predictions and may be preferred because it can be more easily implemented than the BOA model.

## Additional file

**Additional file 1: Table S1.** Standard errors of additive genetic variance ($\upsigma_{\text{a}}^{2}$), litter variance ($\upsigma_{\text{u}}^{2}$), residual variance ($\upsigma_{\text{e}}^{2}$), and heritabilities for each breed for PB and CB performance, and genetic correlations between purebred and CB pigs (${\text{r}}_{\text{pc}}$), estimated for each trait using the BOA^a^, G_A_^b^, and G_B_^c^ models. Description: Standard errors of the estimated variance components, heritabilities, and genetic correlations shown in Table 7.
