# Correction to: Genomic evaluation for a three-way crossbreeding system considering breed-of-origin of alleles

**DOI:** 10.1186/s12711-017-0367-5

**Published:** 2017-12-27

**Authors:** Claudia A. Sevillano, Jeremie Vandenplas, John W. M. Bastiaansen, Rob Bergsma, Mario P. L. Calus

**Affiliations:** 1 Wageningen University & Research Animal Breeding and Genomics, 6700 AH Wageningen, The Netherlands; 2 Topigs Norsvin Research Center, 6640 AA Beuningen, The Netherlands

## Correction to: Genet Sel Evol (2017) 49:75 10.1186/s12711-017-0350-1

After publication of our article [1], we found a typo in the formula to build the genomic relationship matrix using allele frequencies across all genotyped pigs (matrix) and the genomic relationship matrix using breed-specific allele frequencies (matrix), and we noted that the description to this formula is not very clear. In this paper, we describe the corrected formula and provide a clarification.

In the paragraph *Genomic relationship matrix using allele frequencies across all genotyped pigs* (*G*_*A*_ *matrix*), page 4, second column, line 21, a “2” is missing in the formula, i.e., it incorrectly says that **D** is diagonal with $D_{jj} = \frac{1}{{p_{j} \left( {1 - p_{j} } \right)}}$.

Instead, **D** is diagonal with $D_{jj} = \frac{1}{{2p_{j} \left( {1 - p_{j} } \right)}}$.

Similarly, in the paragraph *Genomic relationship matrix using breed*-*specific allele frequencies* (*G*_*B*_ *matrix*), page 4, second column, line 33, a “2” is missing in the formula, i.e., it incorrectly says that **D** ^**B**^ is diagonal with $D_{jj}^{B} = \frac{1}{{p\left( {1 - p_{Bj} } \right)}}$.

Instead, **D** ^**B**^ is diagonal with $D_{jj}^{B} = \frac{1}{{2p_{Bj} \left( {1 - p_{Bj} } \right)}}$.

In the same paragraph, page 4, second column, line 29, **p**_**B**_ is confusingly defined as: “The vector of the frequencies of the counted allele (*p*_*Bj*_). *p*_*Bj*_ was obtained by summing the contribution of each pure breed *j* and the weighted contribution of the CB. The weight was 0.5 for S, and 0.25 for LR and LW.”

For clarification, **p**_**B**_ is the vector of the frequencies of the counted allele at locus *j* (*p*_*Bj*_). For PB, *p*_*Bj*_ was specific for each breed *B* (i.e., S, LR, and LW). For CB, *p*_*Bj*_ was the weighted contribution of each breed. The weights considered for the CB were 0.5 for S, 0.25 for LR and 0.25 for LW.

The original article was corrected.
