# Supplementary material for: Genomic evaluation for a three-way crossbreeding system considering breed-of-origin of alleles
Source: Genet Sel Evol. 2017 Oct 23;49:75. doi: 10.1186/s12711-017-0350-1 (PMC5653471; doi:10.1186/s12711-017-0350-1)
Supplement: Supplementary file 1 — Additional file 1: Table S1. Standard errors of additive genetic variance ($\upsigma_{\text{a}}^{2}$σa2), litter variance ($\upsigma_{\text{u}}^{2}$σu2), residual variance ($\upsigma_{\text{e}}^{2}$σe2), and heritabilities for each breed for PB and CB performance, and genetic correlations between purebred and CB pigs (${\text{r}}_{\text{pc}}$rpc), estimated for each trait using the BOAa, GAb, and GBc models. Description: Standard errors of the estimated variance components, heritabilities, and genetic correlations shown in Table 7. [file 12711_2017_350_MOESM1_ESM.pdf]

Model	Breed	$\sigma^2_{a_{PB}}$	$\sigma^2_{u_{PB}}$	$\sigma^2_{e_{PB}}$	h^2_{PB}	$\sigma^2_{a_{CB}}$	$\sigma^2_{u_{CB}}$*	$\sigma^2_{e_{CB}}$*	h^2_{CB}	r_{pc}
ADG										
BOA	S	374	330	307	0.03	509	170	201	0.07	0.13
	LR	309	252	221	0.03	882				0.18
	LW	196	144	137	0.02	811				0.18
G_A	S	453	327	305	0.03	323	152	209	0.05	0.13
	LR	347	253	222	0.03					0.17
	LW	214	143	137	0.02					0.12
G_B	S	372	327	305	0.03	357	152	209	0.05	0.12
	LR	316	252	222	0.03					0.16
	LW	197	146	137	0.02					0.11
BF										
BOA	S	0.09	0.07	0.07	0.03	0.46	0.17	0.19	0.06	0.12
	LR	0.11	0.08	0.08	0.03	0.77				0.15
	LW	0.09	0.06	0.05	0.02	0.90				0.12
G_A	S	0.11	0.07	0.07	0.03	0.30	0.15	0.19	0.05	0.10
	LR	0.12	0.08	0.08	0.03					0.12
	LW	0.10	0.06	0.05	0.02					0.10
G_B	S	0.09	0.07	0.07	0.03	0.34	0.15	0.20	0.05	0.10
	LR	0.11	0.08	0.08	0.03					0.12
	LW	0.09	0.06	0.05	0.02					0.09
LD										
BOA	S	0.99	0.66	0.53	0.03	2.97	0.93	1.38	0.07	0.12
	LR	0.65	0.44	0.41	0.03	3.72				0.29
	LW	0.45	0.27	0.22	0.02	4.83				0.17
G_A	S	1.19	0.65	0.53	0.03	1.67	0.57	1.42	0.04	0.12
	LR	0.72	0.43	0.41	0.03					0.17
	LW	0.48	0.27	0.22	0.02					0.12
G_B	S	0.98	0.65	0.53	0.03	1.77	0.29	1.42	0.04	0.11
	LR	0.66	0.43	0.41	0.03					0.16
	LW	0.44	0.27	0.22	0.02					0.11

S = Synthetic boar, LR = Landrace (LR), LW = Large White (LW), and CB = three-way crossbred pigs
ADG = average daily gain, BF = back fat, and LD = loin depth
[a]BOA model, model with breed-specific relationship matrices
[b]G_A model, model with genomic relationship matrix by allele frequencies obtained across the genotyped population
[c]G_B model, model with genomic relationship matrix by breed-specific allele frequencies
